# Effects of Air Pollution on the Risk of Congenital Anomalies: A Systematic Review and Meta-Analysis

**DOI:** 10.3390/ijerph110807642

**Published:** 2014-07-31

**Authors:** Esther Kai-Chieh Chen, Denis Zmirou-Navier, Cindy Padilla, Séverine Deguen

**Affiliations:** 1 School of Public Health, EHESP, Sorbonne-Paris-Cité, Rennes, Cedex 35043, France; E-Mails: Esther.Chen@eleve.ehesp.fr (E.K.-C.C.); denis.zmirou@ehesp.fr (D.Z.-N.); cindy.padilla@ehesp.fr (C.P.); 2 Inserm—Unit Research 1085, Research Institute of Environmental and Occupational Health, Rennes, Cedex 35043, France; 3 Lorraine University Medical School, Nancy, Cedex 54052, France

**Keywords:** meta-analysis, congenital anomalies, exposure, air pollution

## Abstract

Congenital anomalies are the main causes of preterm and neonatal mortality and morbidity. We investigated the association between congenital anomalies and mothers’ exposure to air pollution during pregnancy by combining risk estimates for a variety of air pollutants (SO_2_, NO_2_, PM_10_, PM_2.5_, CO and O_3_) and anomaly defect outcomes. Seventeen articles were included in the systematic review and thirteen studies were taken into account in the meta-analysis. Combined estimated were calculated separately according to whether the exposure metric was continuous or categorical. Only one significant combination was; NO_2_ concentrations were significantly associated with coarctation of the aorta (OR = 1.20 per 10 ppb, 95% CI, (1.02, 1.41)). This finding could stem from strong heterogeneity in study designs. Improved exposure assessment methods, in particular more accurate spatial measurements or modeling, standardized definition of cases and of better control of confounders are highly recommended for future congenital anomalies research in this area.

## 1. Introduction

Today, air pollution is a major public health concern. Due to industrial emissions, urbanization and transport of goods and people by fuel-engine motor vehicles, air pollution affects everyone in developed and developing countries. Common pollutants such as nitrogen oxide (NO_2_), sulfur dioxide (SO_2_), particulate matter (PM) and carbon monoxide (CO) have been shown to be associated with several adverse health events such asthma attacks and incidence [1,2], chronic obstructive pulmonary diseases [3], cardio and cerebrovascular conditions and lung cancer [4,5,6], and have been reported to reduce human fertility [7]. Environmental nuisances affect all age groups. However, fetus development, newborns and infants are recognized to be more particularly vulnerable to air pollution [8,9,10]. Exposure of pregnant women is linked with fetal growth retardation, low birth weight [11], preterm birth and neonatal mortality [12]. Maternal exposure to air pollution may be also related to congenital anomalies. However, the evidence is still weak due to the paucity of epidemiological studies.

Congenital anomalies are recognized to be a major risk factor of stillbirth and of neonatal and infant mortality. Worldwide, an estimated 10% deaths under five-years-old children are caused by congenital anomalies [13]. European Surveillance of Congenital Anomalies (EUROCAT), an organization of population-based registries for the surveillance of congenital anomalies in Europe, recorded the perinatal death rate 9.3 per 10,000 births of all congenital anomaly between 2008 and 2012. Among them, 23.7% was due to chromosomal anomalies, 22.6% to congenital heart defects, and 17.2% to nervous system anomalies [14]. About half of all major congenital malformations are of unclear etiology and are suggested to have multifactorial causes, including environmental exposures [15].

In the past decade, the number of studies investigating the association between congenital anomalies and air pollution has increased. The potential impact of environmental exposures to congenital anomalies has been recently reviewed [16,17]. The studies concluded that exposure to NO_2_, SO_2_ and PM_2.5_ increased significantly the risk of congenital heart diseases. Since the last 2011 review, several new studies have been published [18,19,20,21,22,23]. In this setting, updating the literature synthesis may improve our understanding of the relationship between air pollution and congenital anomalies and also of the biological process through which air pollution could lead, directly or indirectly, to these outcomes. We, therefore, conducted a meta-analysis to assess the association between air pollution and the risk of congenital anomalies. We explored whether some subtype of anomalies could be particularly concerned by air pollution. Finally, we discussed hypotheses explaining the different routes by which air pollution might increase the risk of congenital anomalies.

## 2. Methods

The methodology adopted has been described in detail in the previous published review [16]. Meta-analyses were conducted for a minimum number of four individual studies. We separated the data set into two categories according to the metric used for exposure assessment (continuous or categorical). Combined odds ratios were computed in order to contrast the highest with the lowest quartiles of exposure when the individual studies reported categorical exposure metrics. When exposure was expressed as a continuous variable, if quantitative descriptors for air pollutants were available, we also converted effect estimates into ORs contrasting the highest *versus* the lowest exposure categories [24]. Otherwise, risk estimates which had been calculated from continuous exposure metrics were expressed as unit odds ratios, corresponding to an increase of 1 microgram per cubic meter (μg/m^3^) for sulfur dioxide (SO_2_), 10 μg/m^3^ for particulate matter with diameter less than 10 μm (PM_10_) and to an increase of 1 part per million (ppm) for carbon oxide (CO), and 5 part per billion (ppb) for ozone (O_3_) and 10 ppb nitrogen dioxide (NO_2_). Key features and definitions of exposure of each study are detailed in Table 1.

### 2.1. Search Methods

A literature search was conducted in the PUBMED database in order to select articles published between January 2011 and January 2014. The search strategy followed the PRISMA guidelines [25]. The keywords used for this review were (air pollution OR traffic pollution OR outdoor pollution) AND congenital anomalies. We also used the terms “traffic pollution” and “outdoor air pollution”. Searches were restricted to English-language articles. No restriction was put on the geographical location. Abstracts of all studies were then screened manually and excluded if they were not performed on human populations and did not present original data (review articles). Full manuscripts were checked thoroughly. Seven studies were published after 2011; one was not included because its main issue did not deal with the association between congenital anomalies and air pollutants [26]. We also included the eleven articles used in the previous literature synthesis published in 2011. Overall, seventeen articles were included.

### 2.2. Data Extraction

We selected measures from the adjusted models presented in each study. Odds ratios and similar metrics relating outcomes and pollutants were extracted. For cohort studies, we used risk ratios since the two ratios give equivalent results when the outcome is rare. In addition, the period of exposure during the pregnancy has been taken into account since the pregnancy weeks 3–8 constitute the critical window of exposure for embryogenesis; later exposures may not contribute to the etiology of major congenital anomalies [22].

### 2.3. Meta-Analysis

Heterogeneity was assessed for pollutant-congenital anomaly outcomes by using the Cochrane Q-test. Fixed effect models were used when the result of the Q-test gave a heterogeneity *p*-value higher than 0.1. In contrast, random effects models were used for p-values lower than 0.1. Following Higgins *et al.* [27], a low heterogeneity was determined for I^2^ between 25%–50%, moderate between 50%–75%, and high for >75%, where I^2^ is defined as the percentage of variation attributable to heterogeneity. Forest plots were generated to illustrate the combined risk estimates. Statistical analysis was performed using STATA 11 (TX, USA).

**Table 1:** Overview of studies included in the systematic review.

Study	Location	Period	Study Design	Congenital Categories	Exposure Assessment	Exposure Variable	Air Pollutants	Results	Confounders
Gianicolo *et al.* 2014 [23]	Brindisi, Italy	2000–2010	Case-control, individual matching	Congenital heart defects, atrial septal defects	Daily average concentration of pollutants measured by 3 monitoring stations and performed for week 3–8 of gestation	Continuous and categorical	SO_2_ and TSP	Exposure to 90th percentile of SO_2_ increased risk of CHD (*p* = 0.01) and VSD (*p* < 0.05)	No adjusted confounders; cases and controls were matching for gender, socio-economic deprivation and the year of pregnancy
Schembari *et al.* 2013 [22]	Barcelona, Spain	1994–2006	Case-control, no matching	Congenital heart defects, neural tube defects, respiratory system defects, orofacial clefts, digestive system defects, abdominal wall	Daily spatio-temperal exposure estimates over week 3–8 of pregnancy	Continuous	NO_2_, NO_x_, PM_10_, PM_2.5_, PM_coarse_	Statistically significant associations (*p* = 0.05) between NO_2_ and coarctation of the aorta and digestive system defects, and between PM_coarse_ and abdominal wall defects	Maternal age, socio-economic status, year of birth, conception season
Padula *et al.*, 2013 [20]	California, USA	1997–2006	Case-control, no matching	Anotia/microtia, anorectal atresia/stenosis, craniosynostosis, hypospadias degree, diaphragmatic hernia, transverse limb deficiency, intestinal atresia/stenosis, amniotic band syndrome, limb body wall complex, hydrocephaly, longitudinal limb deficiency, esophageal atresia	Residence-based assignments around stations, with daily average values during first two months of were collected; a maximum interpolation radius of 50 km was used	Categorical	NO_2_, NO, CO, O_3_, PM_10_, PM_2.5_	No significant association had been revealed	Maternal ethnicity, education, and early prenatal vitamin use
Padula *et al.* 2013 [19]	California, USA	1997–2006	Case-control, no matching	Congenital heart diseases groups (27 subtypes)	Residence-based assignments around stations, with daily average values during first two months of were collected; a maximum interpolation radius of 50 km was used	Categorical	NO, NO_2_, PM_10_, PM_2.5_, CO, O_3_	No significant association had been revealed	Maternal ethnicity, education, and vitamin use
Agay-Shay K *et al.* 2013 [21]	Tel-Aviv, Israel	2000–2006	Case-control, no matching	Multiple congenital heart, atrial and atrial septal defects, isolated ventricular septal defects	Weekly means of exposures during pregnancy week 3–8 according to the distance from stations to each maternal address	Continuous	NO_2_, SO_2_, PM_10_, PM_2.5_, CO, O_3_	No significant association had been revealed	Infant’s sex, plurality, religion, maternal age, maternal and paternal marital status, maternal and paternal origin, paternal age, and the season of conception
Padula *et al.* 2013 [18]	California, USA	1997–2006	Case-control, no matching	Neural tubes defects (spina bifida and anencephaly), orofacial clefts, gastroschisis	Residence-based assignments around stations, with daily average values during first two months of were collected; a maximum interpolation radius of 50 km was used	Categorical	NO_2_, NO, PM_10_, PM_2.5_, CO, O_3_	No significant association had been revealed	Maternal ethnicity, education, and vitamin use
Dadvand *et al.* 2011 [28]	Northeast of UK	1993–2003	Case-control, frequency matching	Cardiac chambers and connection, cardiac septa, pulmonary and tricupid valves, aortic and mitral valves, great arteries and veins, atrial septal defect, coarctation of aorta, pulmonary valve stenosis, tetralogy of Fallot, ventricular septal defect	Weekly average of pollutants at nearest monitors to maternal residential location	Continuous	SO_2_, NO_2_, CO, PM_10_, O_3_	An association between NO_2_ and congenital heart diseases, ventricular septal defect, cardiac septa malformations and tetralogy of Fallot; and CO exposure to ventricular septal defect, cardiac septa malformations and with congenital pulmonary valve stenosis	Socio-economic status, degree of urbanity, and season of conception; cases and controls were matching for the year of birth
Dadvand *et al.* 2011 [29]	Northeast of UK	1985–1996	Case-control, frequency matching	Coarctation of aorta, tetralogy of fallot, congenital pulmonary value stenosis, atrial septal defect, ventricular septal defect, congenital cardiac chambers and connections, congenital cardia septa, congenital pulmonary and tricuspid valves, congenital aortic and mitral valves, congenital great arteries and veins	Weekly exposure levels by two stage spaiotemporal modeling at each maternal place of residence	Categorical and continuous	SO_2_, black smoke	An association between maternal exposure to black smoke and cardiac chambers and connections (only when using exposure as a continuous variable)	Birth year, socio-economic status, infant sex, season of conception, and degree of urbanity; cases and controls were matching for the year of birth
Marshall *et al.* 2010 [30]	New Jersey, US	1998–2003	Case-control, frequency matching	Cleft lip with cleft palate, cleft palate	Average concentration of exposures at nearest monitor stations (13–20 km)	Categorical	SO_2_, NO_2_, CO, PM_10_, PM_2.5_, O_3_	No significant association had been revealed	Maternal age, ethnicity, smoking and alcohol, and season of conception; cases and controls were matching with maternal residence at birth
Dolk *et al.* 2010 [31]	Wessex, North West Thamas, Oxford and Northern of UK	1991–1999	Cohort	Anomalies of cardiac chambers, transposition of great vessels, malformations of cardiac septa, atrioventricular septal defects, tetralogy of Fallot, malformations of valves, hypoplastice left heart syndrome, great arteries and veins, coarctation of aorta	Annual mean exposure at census level in 1996	Continuous	SO_2_, NO_2_, PM_10_	A significant association between SO_2_ and tetralogy of Fallot, and between PM_10_ and omphalocele	Maternal age, socio-economic deprivation
Hansen *et al.* 2009 [32]	Brisbane, Australia	1998–2004	Case-control, individual matching	Aortic artery and valve defects, atrial septal defects, pulmonary artery and valve defects, ventricular septal defects, conotruncal defects, endocardial cushion and mitral valve defects, cleft lip, cleft palate, cleft lip with cleft palate	Daily average exposures at 18 monitors with the majority located within a 30 km radius of city	Continuous	SO_2_, NO, CO, PM_10_ and O_3_	No significant association had been revealed	Infant sex, birth order, season of birth, maternal age, education, alcohol, and body mass index; cases and controls were matching with mother’s age, marital status, number of previous pregnancies, month of LMP, area-level SES, and distance to pollution monitor
Rankin *et al.*, 2009 [33]	Northern region, UK	1985–1990	Case-control, no matching	Nervous system, congenital heart defects, atrio ventricular septal defects, tetralogy of fallot, hypoplastic left heart, coarctation of aorta, patent ductus arteriosus, ventricular septal defect, respiratory tract, cleft lip and palate, eye, ear, face and neck, digestive system, internal urogenital system, musculoskeletal, miscellaneous	Daily average exposures during the first trimester from monitors within 10 km of maternal residence	Continuous and categorical	SO2, black smoke	A significant association between black smoke and nervous system anomalies.	Birth weight, infant sex, and maternal deprivation
Strickland *et al.* 2009 [34]	Atlanta, USA	1986–2003	Cohort	Tansposition of the great arteries, tetralogy of fallot, pulmonary stenosis and valvar, patent ductus arteriosus, hypoplastic left heart , coarctation of aorta, atrial septal defect, secundum, ventricular septal defect, muscular, ventricular septal defect, permimemnranous, conotruncal defect, Left ventricular outflow tract defect, right ventricular outflow tract defect	Average of daily concentration from one central monitoring station	Continuous	SO_2_, NO_2_, CO, PM_10_, and O_3_	A significant association between PM_10_ and patent ductus arteriosus	No adjusted variables
Hwang *et al.* 2008 [24]	Taiwan	2001–2003	Case-control, no matching	Cleft lip	Monthly average of exposures at 72 stations by using inverse distance weighting method during the first trimester	Continuous	SO_2_, NO_2_, CO, PM_10_ and O_3_	A significant association for first and second month O_3_ exposure	Maternal age, infant sex, plurality ^§^, gestational age, population density, and season of conception
Kim *et al.* 2007 [35]	Seoul, Korea	2001–2004	Birth cohort	Not specified	Residence based average exposure levels at each trimester from nearest monitoring stations	Continuous and categorical	PM_10_	Congenital anomalies were influenced by exposure to PM_10_	Infant sex, birth order, season of birth, maternal age, maternal and paternal education, alcohol, body mass index and maternal weight before delivery
Giloba *et al.* 2005 [36]	Texas, USA	1997–2000	Case-control, frequency matching	Aortic artery and valve defects, atrial septal defects, pulmonary artery and valve defects, ventricular septal defects, conotruncal defects, endo-cardial cushin and mitral valve defects, cleft lip with cleft palate, cleft palate	Average of daily measurements based on the first closest monitor (median distance 8.6–14.2 km)	Categorical	SO_2_, NO_2_, CO, PM_10_ and O_3_	A significant association between exposure to SO_2_ and VSD (*p* < 0.0001), CO and tetralogy of fallot (*p* < 0.0017), PM_10_ and ASD (*p* < 0.0001), SO_2_ with ASD (0.0017)	Maternal age, ethnicity, education, marital status, illness, tobacco use, season ^§^ of conception, plurality, parity, infant sex, prenatal care, and gravidity; cases and control were matching with vital status, year, maternal county of residence at delivery
Ritz *et al.* 2002 [9]	California, USA	1987–1993	Case-control, no matching	Aortic defects, Pulmonary valve, Conotruncal defects, Ventricular septal defects, Multiple cardiac or cleft defect, Syndrome with cardiac or cleft defect, Isolated cleft palate, Isolated cleft lip with/without palate	24 h average measurements every 6 days over duration of pregnancy	Continuous and categorical	CO, O_3_	No significant association had been revealed	Maternal age, ethnicity, education, marital status, illness, tobacco use, season of conception, plurality ^§^, parity, decade of infant’s birth, infant sex, access to prenatal care, time since last pregnancy and birth type

Notes: SO_2_, sodium dioxide; NO, nitrogen oxide; CO, carbon oxide; PM_10_, particulate meter with diameter ≤ 10 μm; PM_2.5_, particulate meter with diameter ≤ 2.5 μm; O_3_, ozone; TSP, total suspended particulate; CHD, congenital heart defects; ASD, atrial septal defects. **^§^** Plurality stands for multiple births (including stillbirth) after one pregnancy.

## 3. Results

Seventeen articles met the inclusion criteria for the systematic review, and the characteristics of each study are shown in Table 1. Three articles published in 2013 were conducted by the same research group [18,19,20]. We considered them as three independent studies because each dealt with different outcomes. Another team also split results into two articles [28,29]. Seven studies were conducted in the United States [9,18,19,20,30,34,36], four in the United Kingdom [28,29,31,33] and only one in Australia [32], in Israel [21], in Italy [23], in South Korea [35], in Spain [22] and in Taiwan [24]. Overall, twelve studies were case-control studies and five were cohort studies. One did not specify congenital subgroups and diagnosed only birth defects (*n* = 14); it was not included in the meta analysis [35].

Most studies used a population-based case-control design, selecting cases from clinical or autopsy reports. Controls were randomly selected from birth registries. Cases included live birth, stillbirth or termination of pregnancy after a congenital anomaly diagnosis. One study collected only newborns with diagnosis of congenital anomalies [23]. Two studies focused specifically on orofacial defects [24,30] and therefore included only cases with a diagnosis of cleft lip with or without cleft palate. Definitions of cases with single or multiple congenital defects and criteria for splitting them into sub-groups varied across studies. Cases definition used in the studies published after 2010 were mainly coded according to the International Classification of Diseases, version 9 or 10 (ICD codes). Cardiovascular anomalies were the most frequently investigated defects, followed by neuron system.

Mother residence concentration estimates from air quality monitoring networks were frequently used for exposure assessment. Average concentrations of pollutants during the first or the first two months of pregnancy were calculated from the nearest monitors. The distance from monitors to maternal residence varied among studies, from 10 km to a maximum of 50 km. Classical air pollutants were measured, *i.e.*, SO_2_, NO_2_, PM_10_, PM_2.5_, CO and O_3_ in most studies. Total suspended particulate (TSP), NO, NO_x_ and black smoke were assessed in a small number of studies. Pollutant concentration distributions are shown in Table 2.

We conducted the meta-analysis for 21 combinations of air pollutants and congenital anomalies when at least four studies were available for the same combination. Heterogeneity tests (the Q-test) indicated four combinations with high values of I^2^, for which random effects models were applied. Heterogeneity varied between 0% and 93.4%, which indicated that the measurement methods, sample property and characteristics varied among and within different congenital groups.

**Table 2:** Exposure distribution in studies included in the meta analysis.

Studies	Case		Control		Total
	Congenital Defects	Air Pollutants	Congenital Defects	Air Pollutants	
Gianicolo *et al.* 2014 [23]	CHD	SO_2_, mean 2.9 μg/m^3^	CHD	SO_2_, mean 2.8 μg/m^3^	-
	VSD	SO_2_, mean 3.2 μg/m^3^	VSD	SO_2_, mean 2.8 μg/m^3^	
Schembari *et al.* 2013 [22]	-	IQR: NO_2_, 12.7 μg/m^3^ PM_10_, 2.8 μg/m^3^	-	NO_2_, IQR 11.8 μg/m^3^	-
	-		-	PM_10_, IQR 3.0 μg/m^3^	
Agay-Shay *et al.* 2013 [21]	-	-	-	-	Minimum, median, maxmum: SO_2_ 0.33 ppb, 2.1 ppb, 51.4 ppb; CO, 0.15 ppm, 0.9 ppm, 13.5 ppm; NO_2_ 0.2 ppb, 23.1 ppb, 104.5 ppb; O_3_ 0.45 ppb, 26.5 ppb, 128 ppb; PM_10_, 3.8 μg/m^3^, 43, 3183.4 μg/m^3^
Padula *et al.* 2013 [18]	-	-	-	-	CO, Q1 0.13–0.39 ppm, Q4 0.72–1.37 ppm; NO_2_, Q1 2.4–13.36 ppb, Q4 20.54–638.94 ppb; O_3_, Q1 10.49–29.05 ppb, Q4 62.65–91.92 ppb; PM_10_, Q1 7.9–25.24 μg/m^3^, Q4 44.09–95.32 μg/m^3^
Dadvand *et al.* 2011 [29]	-	-	-	-	Percentile 25- percentile 75: CO, 0.39–0.64 mg/m^3^; NO_2_, 29.2–38.4 μg/m^3^; NO, 13.3–32.5 μg/m^3^; O_3_, 33.2–42.4 μg/m^3^; PM_10_, 20.5–30.2 μg/m^3^
Dadvand *et al.* 2011 [28]	-	-	-	-	Percentile 25–percentile 75: SO_2_, 17.6–31.2 μg/m^3^
Marshall *et al.* 2010 [30]	-	Mean: PM_10_, 28.7 μg/m^3^ NO_2_, 2.4E−2 ppm SO_2_, 5.3E−3 ppm O_3_, 2.5E−2 ppm CO, 0.83 ppm	-	Mean: PM_10_, 28.1 μg/ m^3^ NO_2_, 2.4E−2 ppm SO_2_, 5.1E−3 ppm O_3_, 2.5E−2 ppm CO, 0.85 ppm	-
Dolk *et al.* 2010 * [31]	-	-	-	-	Percentile 10, median, percentile 90: SO_2_, 3.87 μg/m^3^, 7.86 μg/m^3^, 14.99 μg/m^3^ NO_2_, 21.48 μg/m^3^, 35.11 μg/m^3^, 47.78 μg/m^3^ PM_10_, 18.84 μg/m^3^, 21.97 μg/m^3^, 26.4 μg/m^3^
Hansen *et al.* 2009 [32]	-	-	-	-	Minmum, mean, maxmum: SO_2_, 0, 1.5 ppb, 7.1 ppb; CO, 0.02 ppm, 1.1 ppm, 7.0 ppm; NO_2_, 1.4 ppb, 8.2 ppb, 22.7 ppb; O_3_, 4.3 ppb, 25.8 ppb, 54.4 ppb; PM_10_, 4.4 μg/m^3^, 18.0 μg/m^3^, 151.7 μg/m^3^
Rankin *et al.* 2009 [33]	-	SO_2_ Q1–Q3, 2.7–4.4 μg/m^3^	-	-	-
Strickland *et al.* 2009 * [34]	-	-	-	-	IQR: SO_2_, 4.0 ppb; CO, 0.3 ppm; NO_2_, 5.7 ppb; O_3_, 29.9 μg/m^3^; PM_10_, 14.2 μg/m^3^
Hwang *et al.* 2008 [24]	-	-	-	-	Minmum, median, maxmum: O_3_, 16.7 ppb, 26.8 ppb, 45 ppb; CO, 25 pphm, 62 pphm, 277 pphm; NO_x_, 1.0 ppb, 20.2 ppb, 44.2 ppb; PM_10_, 20.8 μg/m^3^, 57.2 μg/m^3^, 78.1 μg/m^3^
Gioboa *et al.* 2005 [36]	-	-	-	-	CO, Q1 < 0.4 ppm, Q4 ≥ 0.7 ppm; NO_2_, Q1 <1.3 pphm, Q4 ≥ 2.1 pphm; O_3_, Q1 < 1.8 pphm, Q4 ≥ 3.1 pphm; SO_2_, Q1 < 1.3 ppb, Q4 ≥ 2.7 ppb; PM_10_, Q1 < 19.5 μg/m^3^, Q4 ≥ 29 μg/m^3^

Notes: CHD, congenital heart defects; VSD, ventricular heart defects; IQR, interquartile range.; Q1, quartile 1; Q2, quartile 2; Q3, quartile 3; Q4, quartile 4; * indicated cohort studies, others were designed as case-control studies; “-” no information.

**Figure 1:**
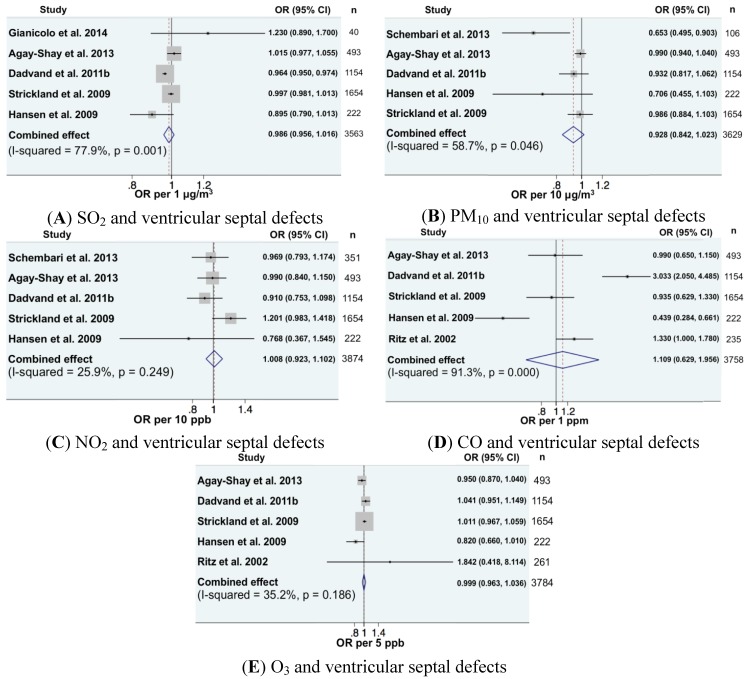
Forest plots for combinations of ventricular septal defects and pollutant (as a continuous variable). The size of each square represents the weight that contributes to the combined effect, respectively for: (**A**) SO_2_; (**B**) PM_10_; (**C**) NO_2_; (**D**) CO; and (**E**) O_3_.

**Figure 2:**
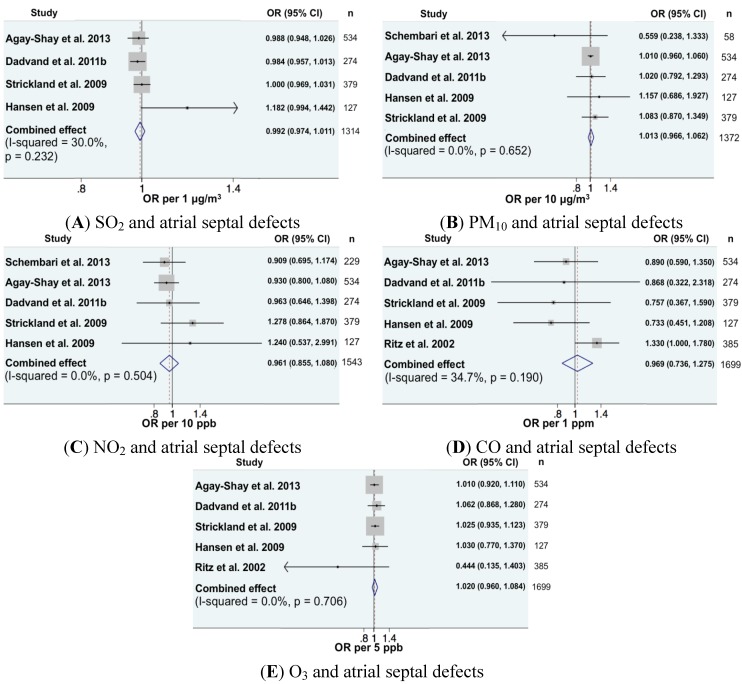
Forest plots for combinations of atrial septal defects and pollutant (as a continuous variable). The size of each square represents the weight that contributes to the combined effect, respectively for: (**A**) SO_2_; (**B**) PM_10_; (**C**) NO_2_; (**D**) CO and (**E**) O_3_.

**Figure 3:**
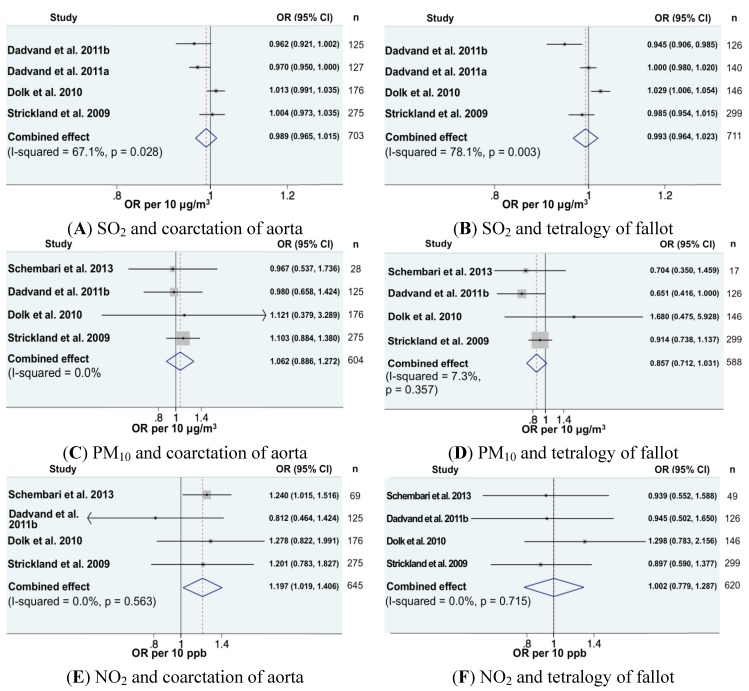
Forest plots for combinations of two cardiac anomalies (coarctation of aorta and tetralogy of fallot) and pollutant. The size of each square represents the weight that contributes to the combined effect. (**A**), (**C**), (**E**) were combined effects of coarctation of aorta and SO_2_, PM_10_ and NO_2_. (**B**), (**D**) and (**F**) were combined effects of tetralogy of fallot and SO_2_, PM_10_ and NO_2_.

Regarding cardiovascular anomalies, 16 combinations of pollutants-cardiac anomalies could be included in the meta-analysis (Figure 1, Figure 2 and Figure 3). In all, exposure was expressed as continuous variables. We found a significantly increased meta-OR for exposure to NO_2_ and the risk of coarctation of aorta (OR per 10 ppb = 1.20, 95% CI (1.02, 1.41)), which is in accord with the previous meta-analysis [16]. For all other combinations, the combined effects were close to one and not significant.

**Figure 4:**
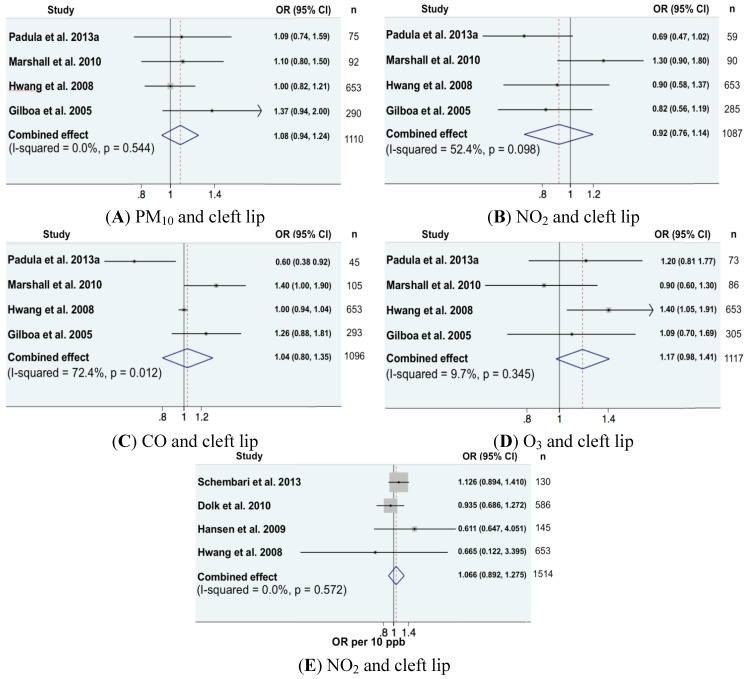
Forest plots for a variety of pollutants and risk of orofacial defects. (**A**), (**B**), (**C**) and (**D**) showed combined effects of PM_10_, NO_2_, CO and O_3_; (**E**) was the combined effect of NO_2_ and cleft lip from continuous exposure risk estimates.

For oro-facial clefts, seven articles were included in the meta-analysis, with one study exhibiting a statistically significant association [24]: the OR comparing the 4th with the 1st quartiles of the exposure distribution was equal to 1.40 (95% CI (1.05, 1.91)). The meta-analysis found no significant association for the four other pollutants (Figure 4A–D). In most cases, the studies on cleft lips, for which exposure was expressed on a continuous scale, were less than four, except for those exploring the effect of exposure to NO_2_ whose result is presented in Figure 4E also showing no significant association.

## 4. Discussion 

Congenital anomalies are the leading cause of neonatal mortality. The prevalence in France reported by the national health surveillance institute is equal to 3.3% [37]. In this systematic review, we assessed the effects of air pollution on the risk of congenital anomalies based on the epidemiologic literature. We compared 21 combinations of air pollutants and congenital anomalies types and only one significant result was revealed. The input data of the 21 combinations were different; it is improbable that the number of statistical tests performed explains the result. But, as all significant results, we cannot exclude that it is a chance finding. The combined risk of coarctation of aorta was found significantly associated with NO_2_ (OR per 10 ppb = 1.20, 95% CI (1.02, 1.41)). Our meta-analysis did not reveal any other significant association for cardiac anomalies and oral-facial defects, which is in coherence with the previous meta-analysis published in 2010 by Vrijheid *et al.* [16].

However, these authors revealed a significantly increased risk between exposure to NO_2_, SO_2_ and the tetralogy of fallot (OR per 10 ppb NO_2_ = 1.25, 95% CI (1.02–1.51); and OR per 1 ppb SO_2_ = 1.04, 95% CI (1.00–1.08), respectively) and coarctation of aorta (OR per 10 ppb NO_2_ = 1.20, 95%CI (1.00–1.44); OR per 1 ppb SO_2_= 1.04; 95% CI (1.00–1.08)). We did not undertake the meta-analysis for these combinations because the number of studies was small. In the present work, we chose to realize two-separate meta-analyses according to the type (continuous or categorical) of the exposure variable, not to introduce too much heterogeneity in the meta-estimates, whereas Vrijheid *et al.* converted all continuous to categorical variables in order to increase their statistical power.

The present meta-analysis has several limitations. The first one deals with the sample size. Most of the combined effects were computed with about five studies; we did not try to compute combined effects for less than four studies. There was, however, a large total number of cases included in the meta-analysis (varying from 588 to 3874 according to the air pollutant-congenital anomaly combination), that provided enough statistical power to reveal significant associations. The numbers of cases and controls of each study are reported in Table 3, Table 4, Table 5, Table 6 and Table 7. We calculated the statistical power (a fixed alpha-risk = 5%) for the smallest sample size (Table 7) and the highest (Table 5 and Table 6). With a statistical power equal to 90%, we will detect a statically significant health effect equal to 18% and 25% from the smallest and the highest sample size, respectively; note that, due to the small number of cases counted in the highest sample size (about 600 cases among a population of about 1.5 million because of the cohort studies include in the meta analysis), a smaller effect could be found significant from the smallest sample size. The statistical power will fall to 70% and 60% (respectively, in the smallest and highest samples sizes) to reveal a significant increase of the risk equal to 10%.

Secondly, we found differences in the methodologies adopted in the 17 individual studies included in the present review, which make difficult the comparisons and assessment of the combined effects. Certain studies relied on measurement of air quality at the birth residence [9,28,29,30,31,32,33,36], which is not necessarily the address during early pregnancy (the critical window time) and hence may lead to misclassification of exposure for several pregnant women. In addition, without distance-weighted calculation in the exposure assessments, the range of distance between maternal place and the nearest monitoring station may produce uncertain exposure estimates and thus lead also to exposure misclassification. 

**Table 3:** Numbers of ventricular septal defects cases and number of controls in studies included in the meta analysis for 5 pollutants: SO_2_, PM_10_, NO_2_, CO, O_3._

Numbers of cases and controls exposed under various pollutants among different studies	Gianicolo *et al.* 2014 [23]		Schembari *et al.* 2013 [22]		Agay-Shay *et al.* 2013 [21]		Dadvand *et al.* 2011 [29]		Strickland *et al.* 2009 [34]		Hansen *et al.* 2009 [32]		Ritz *et al.* 2002 [9]		Total Number	
	**case**	**control**	**case**	**control**	**case**	**control**	**case**	**control**	**case**	**control**	**case**	**control**	**case**	**control**	**case**	**control**
SO_2_	40	150	-	-	493	130,402	1154	4616	1654	713,846	222	1110	235	9049	3798	859,173
PM_10_	-	-	106	903	493	130,402	1154	4616	1654	713,846	222	1110	235	9049	3864	859,926
NO_2_	-	-	351	2869	493	130,402	1154	4616	1654	713,846	222	1110	-	-	3874	852,843
CO	-	-	-	-	493	130,402	1154	4616	1654	713,846	222	1110	-	-	3523	849,974
O_3_	-	-	-	-	493	130,402	1154	4616	1654	713,846	222	1110	-	-	3523	849,974

**Table 4:** Numbers of atrial septal defects cases and number of controls in studies included in the meta analysis for 5 pollutants: SO_2_, PM_10_, NO_2_, CO, O_3._

Numbers of cases and controls exposed under various pollutants among different studies	Schembari *et al.* 2013 [22]		Agay-Shay *et al.* 2013 [21]		Dadvand *et al.* 2011 [29]		Strickland *et al.* 2009 [34]		Hansen *et al.* 2009 [32]		Ritz *et al.* 2002 [9]		Total Number	
	**case**	**control**	**case**	**control**	**case**	**control**	**case**	**control**	**case**	**control**	**case**	**control**	**case**	**control**
SO_2_	-	-	534	130,402	274	1096	379	715,121	127	635	-	-	1314	847,254
PM_10_	106	903	534	130,402	274	1096	379	715,121	127	635	-	-	1420	848,157
NO_2_	229	2869	534	130,402	274	1096	379	715,121	127	635	-	-	1543	850,123
CO	-	-	534	130,402	274	1096	379	715,121	127	635	385	3000	1699	850,254
O_3_	-	-	534	130,402	274	1096	379	715,121	127	635	385	3000	1699	850,254

**Table 5:** Numbers of coarctation of aorta cases and number of controls in studies included in the meta analysis for 3 pollutants: SO_2_, PM_10_, NO_2_.

Numbers of cases and controls exposed under various pollutants among different studies	Schembari *et al.* 2013 [22]		Dadvand *et al.* 2011 [29]		Dadvand *et al.* 2011 [28]		Dolk *et al.* 2010 [31]		Strickland *et al.* 2009 [34]		Total Number	
	**case**	**control**	**case**	**control**	**case**	**control**	**case**	**control**	**case**	**control**	**case**	**control**
SO_2_	-	-	125	500	127	508	176	759,817	275	715225	703	1,476,050
PM_10_	28	890	125	500	-	-	176	759,817	275	715225	604	1,476,432
NO_2_	69	2869	125	500	-	-	176	759,817	127	715225	497	1,478,411

**Table 6:** Numbers of tetralogy of fallot cases and number of controls in studies included in the meta analysis for the 3 pollutants: SO_2_, PM_10_, NO_2_.

Numbers of cases and controls exposed under various pollutants among different studies	Schembari *et al.* 2013 [22]		Dadvand *et al.* 2011 [29]		Dadvand *et al.* 2011 [28]		Dolk *et al.* 2010 [31]		Strickland *et al.* 2009 [34]		Total Number	
	**case**	**control**	**case**	**control**	**case**	**control**	**case**	**control**	**case**	**control**	**case**	**control**
SO_2_	-	-	126	504	140	560	146	759,817	299	715,201	711	1,476,082
PM_10_	17	890	126	504	-	-	146	759,817	299	715,201	588	1,476,412
NO_2_	49	2650	126	504	-	-	146	759,817	299	715,201	620	1,478,172

**Table 7:** Numbers of cleft lip cases and number of controls in studies included in the meta analysis for 4 pollutants: PM_10_, NO_2_, CO, O_3._

Numbers of cases and controls exposed under various pollutants among different studies	Padula *et al.* 2013 [18]		Marshall *et al.* 2010 [30]		Hwang *et al.* 2008 [24]		Giloba *et al.* 2005 [36]		Total Number	
	**case**	**control**	**case**	**control**	**case**	**control**	**case**	**control**	**case**	**control**
PM_10_	75	200	92	12,925	653	6530	290	3450	1110	23,105
NO_2_	59	205	92	12,925	653	6530	285	3237	1089	22,897
CO	45	157	92	12,925	653	6530	293	3309	1083	22,921
O_3_	73	201	92	12,925	653	6530	305	3594	1123	23,250

Different classifications of congenital anomalies also yield some heterogeneity. Specific definition of defects could ease detection of significant associations. Ventricular septal defects are the most common congenital subgroups that were studied and a variety of classifications were found. For example, ventricular septal defects were classified into three subgroups (ventricular septal defects-permembranous, muscular and conov) in the Padula *et al.* study [19], according to the classification proposed by Botto *et al*. [38], whereas in others they used two subgroups (ventricular septal defect, muscular and permembranous) [31] or only one global group [21,29,32,36]. Another example is the subcategory “conotruncal defect”: four studies used this outcome category to include other cardiovascular malformations, such as “tetralogy of fallot”, “transposition of the great arteries”, truncus arteriosus communis, “double outlet right ventricle” or “aorticopulmonary window” [9,31,32,36]; while, tetralogy of fallot and transposition of the great arteries were considered as an event in most other studies. 

Confounding factors included in the individual studies are an additional problem. Few confounders, such as smoking [39], parental occupation [40], maternal age [41] and season conception [42] have been addressed in congenital anomalies research. Season and maternal age at conception were the most frequent confounders considered in the studies included in the present work. Seasonal variations of congenital anomalies incidence have been well described, with a higher risk in summer than in winter [42]. The association between maternal age and the risk of non-genetic congenital anomalies is still unclear. Previous studies analyzing the EUROCAT database found that teenage mothers were at higher risk but not older mothers (35–44 years). However, among European countries, the maternal age patterns are less clear. This suggests the influence of social factors, exposures or living habits, which might be associated with maternal age [41]. The prevalence of congenital anomalies is related to the social status, with higher values in deprived categories [43]. Now, few studies have addressed socioeconomic status/deprivation as confounders or effect modifiers [22,27,28,36]. One study matched cases and controls according to a neighborhood socioeconomic deprivation index but without considering other confounders in the statistical analysis [23]. One may consider that socioeconomic deprivation would constitute a good approximation of well-known risk factors of congenital abnormalities such as smoking habits or educational level. The wide range of confounders considered in the individual studies included in the present review might introduce heterogeneity when combining the data. Because of these limitations, this meta-analysis could detect only few significant associations between air pollution and birth defects; however it does not mean that the hypothesis should be definitively disregarded. 

The different methods used in the studies, such as case and control definition, exposure assessment and confounding factors as all mentioned earlier, could damage the quality level of each study included in the meta-analysis and consequently the quality of the combined estimates. Assessing the quality of studies is important to understand properly each study to be used in meta analysis. It would be interesting to use the Newcastle-Ottawa Scale (NOS) [44] followed by quality score analysis as recommended by Detsky *et al.* [45] in order to assess the quality of each study. Then, including only studies with the highest quality score in the meta-analyses, we could measure more precisely the impact of the study quality on the point estimates. However, due to the limited number of studies in our meta-analyses, it was not possible to conduct this procedure.

Language selection may also bias the data basis. Non-English publications of relevant articles may have been ignored. Moreover, the risk of publication bias is inherent in systematic literature review. Unpublished results (probably, more likely to bear not significant results and the grey literature, which is not available on open sources) may distort the meta-analysis findings. So far, the two meta-analyses (the one published in 2010 and the present one) tend to suggest an adverse effect of air pollution on at least one type of birth defects, and this call for further studies in order to confirm the finding. We failed to assess publication bias by using funnel plots. According to the recommendations from Cochrane Handbook for Systematic Reviews of Interventions [46], there should be at least 10 studies in the meta-analysis to distinguish real asymmetry. 

Air pollutants could directly exert adverse effects as pro-oxidants binding to lipid and proteins, therefore promoting oxidative stress and the production of free radicals, a process that may elicit a variety of diseases or defects [47]. This oxidative stress caused by air pollution during pregnancy has been pointed out in some studies [48,49]. In addition, there is recent evidence that air pollutants can contribute to epigenetic changes, including alteration of DNA methylation [50]. MicroRNA has been also studied with regard of the environmental changes and there is evidence that microRNA expression and regulation may be affected by environmental exposures, such as air pollution, smoking and heavy metal accumulation [51]. Such epigenetic modifications during pregnancy could impair normal embryo development and lead to birth defects. 

## 5. Conclusions

Air pollution is a universal issue. Therefore, a small increase in risks may lead to serious public health problems. Congenital anomalies are the main causes of preterm and neonatal mortality and morbidity. Meta-analysis is an appropriate tool to enhance statistical power in the analysis of weak associations. It might shed new light on the association between air pollution and congenital anomalies insofar as new studies are conducted that overcome the limitations discussed in the present literature review. Improved exposure assessment methods, in particular more accurate spatial measurements or modeling, standardized definition of cases and accommodation of known or putative confounders are highly recommended for future congenital anomalies research on the effect of air pollution.

## Supplementary Files

Supplementary File 1
