# Supplementary material for: Effects of Air Pollution on the Risk of Congenital Anomalies: A Systematic Review and Meta-Analysis
Source: Int J Environ Res Public Health. 2014 Jul 31;11(8):7642–68. doi: 10.3390/ijerph110807642 (PMC4143824; doi:10.3390/ijerph110807642)
Supplement: Supplementary File 1 [file ijerph-11-07642-s001.pdf]

Supplementary Information

Effects of Air Pollution on the Risk of Congenital Anomalies: A Systematic Review and Meta-Analysis

Table S1. Numbers of cases and controls of congenital heart defects in studies included in the meta analysis.

Articles	Defects	Exposure	Case	Control
Gianicolo *et al.*, 2014 [1]	VSD	SO_2	40	150
Schembari *et al.*, 2013 [2]	VSD	NO_2	351	2869
	VSD	PM_{10}	106	903
	ASD	NO_2	229	2869
	ASD	PM_{10}	58	903
	COA	PM_{10}	28	890
	COA	NO_2	69	2869
	TF	PM_{10}	17	890
	TF	NO_2	49	2650
Agay-Shay *et al.*, 2013 [3]	VSD	SO_2	493	130,402
	VSD	PM_{10}	493	130,402
	VSD	NO_2	493	130,402
	VSD	CO	493	130,402
	VSD	O_3	493	130,402
	ASD	SO_2	534	130,402
	ASD	PM_{10}	534	130,402
	ASD	NO_2	534	130,402
	ASD	CO	534	130,402
	ASD	O_3	534	130,402
Dadvand *et al.*, 2011 [4]	VSD	SO_2	1154	4616
	VSD	PM_{10}	1154	4616
	VSD	NO_2	1154	4616
	VSD	CO	1154	4616
	VSD	O_3	1154	4616
	ASD	SO_2	274	1096
	ASD	PM_{10}	274	1096
	ASD	NO_2	274	1096
	ASD	CO	274	1096
	ASD	O_3	274	1096
	COA	SO_2	125	500
	COA	PM_{10}	125	500
	COA	NO_2	125	500
	TF	SO_2	126	504
	TF	PM_{10}	126	504
	TF	NO_2	126	504

Table S1. *Cont.*

Articles	Defects	Exposure	Case	Control
Dadvand *et al.*, 2011 [5]	COA	SO_2	127	508
	TF	SO_2	140	560
Dolk *et al.*, 2010 [6]	COA	SO_2	176	759,817
	COA	PM_{10}	176	759,817
	COA	NO_2	176	759,817
	TF	SO_2	146	759,817
	TF	PM_{10}	146	759,817
	TF	NO_2	146	759,817
Hansen *et al.*, 2009 [7]	VSD	SO_2	222	1110
	VSD	PM_{10}	222	1110
	VSD	NO_2	222	1110
	VSD	CO	222	1110
	VSD	O_3	222	1110
	ASD	SO_2	127	635
	ASD	PM_{10}	127	635
	ASD	NO_2	127	635
	ASD	CO	127	635
	ASD	O_3	127	635
Strickland *et al.*, 2009 [8]	VSD	SO_2	1654	713,846
	VSD	PM_{10}	1654	713,846
	VSD	NO_2	1654	713,846
	VSD	CO	1654	713,846
	VSD	O_3	1654	713,846
	ASD	SO_2	379	715,121
	ASD	PM_{10}	379	715,121
	ASD	NO_2	379	715,121
	ASD	CO	379	715,121
	ASD	O_3	379	715,121
	COA	SO_2	275	715,225
	COA	PM_{10}	275	715,225
	COA	NO_2	275	715,225
	TF	SO_2	299	715,201
	TF	PM_{10}	299	715,201
	TF	NO_2	299	715,201
Ritz *et al.*, 2002 [9]	VSD	CO	235	9049
	VSD	O_3	235	9049
	ASD	CO	385	3000
	ASD	O_3	385	3000

Table S2. Numbers of cases and controls of cleft lip defects in studies included in the meta analysis.

Articles	Exposure	Case	Control
Padula *et al.*, 2013 [10]	PM_{10}	75	200
	NO_2	59	205
	CO	45	157
	O_3	73	201
Marshall *et al.*, 2010 [11]	PM_{10}	92	12,925
	NO_2	90	12,925
	CO	105	12,925
	O_3	86	12,925
Hwang *et al.*, 2008 [12]	PM_{10}	653	6530
	NO_2	653	6530
	CO	653	6530
	O_3	653	6530
Giloba *et al.*, 2005 [13]	PM_{10}	290	3450
	NO_2	285	3237
	CO	293	3309
	O_3	305	3594

References

1. Gianicolo, E.A.; Mangia, C.; Cervino, M.; Bruni, A.; Andreassi, M.G.; Latini, G. Congenital anomalies among live births in a high environmental risk area—A case-control study in Brindisi (southern Italy). *Environ. Res.* **2014**, *128*, 9–14.
2. Schembari, A.; Nieuwenhuijsen, M.J.; Salvador, J.; de Nazelle, A.; Cirach, M.; Dadvand, P.; Beelen, R.; Hoek, G.; Basagaña, X.; Vrijheid, M. Traffic-related air pollution and congenital anomalies in Barcelona. *Environ. Health Perspect.* **2014**, *122*, 317–323.
3. Agay-Shay, K.; Friger, M.; Linn, S.; Peled, A.; Amitai, Y.; Peretz, C. Air pollution and congenital heart defects. *Environ. Res.* **2013**, *124*, 28–34.
4. Dadvand, P.; Rankin, J.; Rushton, S.; Pless-Mulloli, T. Ambient air pollution and congenital heart disease: A register-based study. *Environ. Res.* **2011b**, *111*, 435–441.
5. Dadvand, P.; Rankin, J.; Rushton, S.; Pless-Mulloli, T. Association between maternal exposure to ambient air pollution and congenital heart disease: A register-based spatiotemperal analysis. *Amer. J. Epidemiol.* **2011a**, *173*, 171–182.
6. Dolk, H.; Armstrong, B.; Lachowycz, K.; Vrijheid, M.; Rankin, J.; Abramsky, L.; Boyd, P.A.; Wellesley, D. Ambient air pollution and risk of congenital anomalies in England, 1991–1999. *Occup. Environ. Medicine* **2010**, *67*, 223–227.
7. Hansen, C.A.; Barnett, A.G.; Jalaludin, B.B.; Morgan, G.G. Ambient air pollution and birth defects in Brisbane, Australia. *PLoS One* **2009**, *4*, doi:10.1371/journal.pone.0005408

8. Strickland, M.J.; Klein, M.; Correa, A.; Reller, M.D.; Mahle, W.T.; Riehle-Colarusso, T.J.; Botto, L.D.; Flanders, W.D.; Mulholland, J.A.; Siffel, C,; *et al.* Ambient air pollution and cardiovascular malformations in Atlanta, Georgia, 1986–2003. *Amer. J. Epidemiol.* **2009**, *169*, 1004–1014.
9. Ritz, B.; Yu, F.; Fruin, S.; Chapa, G.; Shaw, G.M.; Harris, J.A. Ambient air pollution and risk of birth defects in southern California. *Amer. J. Epidemiol.* **2002**, *155*, 17–25.
10. Padula, A.M.; Tager, I.B.; Carmichael, S.L.; Hammond, S.K.; Lurmann, F.; Shaw, G.M. The association of ambient air pollution and traffic exposures with selected congenital anomalies in the San Joaquin Valley of California. *Amer. J Epidemiol.* **2013a**, *177*, 1074–1085
11. Marshall, E.G.; Harris, G.; Wartenberg, D. Oral cleft defects and maternal exposure to ambient air pollutants in New Jersey. *Birth Defects Res. Pt. A* **2010**, *88*, 205–215.
12. Hwang, B.F.; Jaakkola, J.J.K. Ozone and other air pollutants and risk of oral clefts. *Environ. Health Perspect.* **2008**, *116*, 1411–1415.
13. Giloba, S.M.; Mendola, P.; Olshan, A.F.; Langlois, P.H.; Savitz, D.A.; Loomis, D.; Herring, A.H.; Fixler, D.E. Relation between ambient air quality and selected birth defects, seven county study, Texas, 1997–2000. *Amer. J. Epidemiol.* **2005**, *162*, 238–252.

© 2014 by the authors; licensee MDPI, Basel, Switzerland. This article is an open access article distributed under the terms and conditions of the Creative Commons Attribution license (http://creativecommons.org/licenses/by/3.0/).
